# Do prostate cancer-related mobile phone apps have a role in contemporary prostate cancer management? A systematic review by EAU young academic urologists (YAU) urotechnology group

**DOI:** 10.1007/s00345-020-03197-w

**Published:** 2020-04-22

**Authors:** Enakshee Jamnadass, Bhavan Prasad Rai, Domenico Veneziano, Theodoros Tokas, Juan Gomez Rivas, Giovanni Cacciamani, Bhaskar Somani

**Affiliations:** 1 University Hospital Southampton NHS Trust, Southampton, UK; 2 Freeman Hospital, Newcastle, UK; 3 Department of Urology and Renal Transplantation, Bianchi-Melacrino-Morelli Hospital, Reggio Calabria, Italy; 4 Department of Urology, Hospital Universitario La Paz, Madrid, Spain; 5 Department of Urology, USC Urology Institute, University of Southern California, Los Angeles, CA USA; 6 Tirol Kliniken, Milser Strasse 10, Hall in Tirol, Austria

**Keywords:** Prostate cancer, Mobile phone applications, Social media

## Abstract

**Aims and objectives:**

To review the available literature regarding the use of prostate cancer-related mobile phone applications (PCA).

**Materials and methods:**

The search was for English language articles between inceptions of databases to June 2019. Medline, EMBASE, Cochrane Library, CINAHL and Web of Science were searched. Full-text articles were reviewed, and the following data were extracted to aid with app analysis: name of application, developer, platform (Apple App Store or Google Play Store) and factors assessed by the article.

**Results:**

The search yielded 1825 results of which 13 studies were included in the final review. 44 PCAs were identified from the data collected of which 59% of the PCAs had an educational focus. 11 apps were inactive and 5 weren’t updated within the last year. Five studies focused on the development and testing of apps (MyHealthAvatar, CPC, Rotterdam, Interaktor, NED). Two studies evaluated the readability of PCAs. Most PCAs had a reading level greater than that of the average patient. Two studies evaluated the quality and accuracy of apps. Majority of PCAs were accurate with a wide range of information. The study reported most PCAs to have deficient or insufficient scores for data protection. Two studies evaluated the accuracy of Rotterdam, CORAL and CPC risk calculators. Rotterdam was the best performer.

**Conclusions:**

PCAs are currently in its infancy and do require further development before widespread integration into existing clinical practise. There are concerns with data protection, high readability standards and lack of information update in current PCAs. If developed appropriately with responsible governance, they do have the potential to play important roles in modern-day prostate cancer management

## Introduction

Information Communication Technology (ICT) is an integral part of modern-day healthcare delivery in domains such as education, research, operational efficiency and data management [1]. In prostate cancer with changing diagnostic and therapeutic paradigms, there is likely to be reliance on technology for the delivery of cost-effective, high-quality cancer care [2]. Mobile phone application (apps) is software with specific, limited function, which is designed for use on a mobile device [3]. It has been suggested mobile phone apps have the potential to increase patient awareness, be adjuncts to traditional clinical evaluation strategies and can also facilitate research development and delivery [4]. The two most popular platforms, from which users can download apps, are the Google Play Store, and the Apple App store. Over 2 million apps are available on these platforms [5]. There are over 5.5 billion smartphone users worldwide [6], and it is estimated that the average user spends over 3.5 h on their mobile device every day [7]. Furthermore, users spend 89% of their media time on mobile apps [8]. In the United Kingdom, a reported 75% of people go online for health information. Additionally, 70% of patients aged over 50 want to use digital healthcare services [9]. The market for healthcare-related apps is growing, and it is suggested that around 200 healthcare apps are added daily [9].

Prostate cancer is the most common cancer in males, in the UK (second most common in men, worldwide) and, according to the American Cancer Society, 1 in 9 men will be diagnosed with prostate cancer during their lifetime [10, 11]. Furthermore, the incidence of prostate cancer is increasing and is projected to rise in the UK by 12% between 2014 and 2035 [10]. Given the significance of prostate cancer worldwide, and the increasing usage of healthcare apps within patient populations, we aim to systematically review the available literature regarding the availability and usage of prostate cancer-related mobile phone apps (PCA). We also look at the type of app, its content, rating and their real-world application.

## Materials and methods

### Selection criteria

This review included studies that explored and evaluated various aspects of PCAs, as well as their current and potential applications in the screening, prevention or management of prostate cancer.

### Inclusion and exclusion criteria

Inclusion criteria:

I. English-language oncological papers with a focus on prostate cancer.
II. Studies reporting on mobile phone apps for prostate cancer.

Exclusion criteria:

I. Literature reviews, grey literature, editorials, letters, and other ‘comment’ pieces.
II. Studies on prostate cancer not related to apps.
III. Studies relating to apps which are unpublished or unreleased.

### Search strategy

**Fig. 1:**
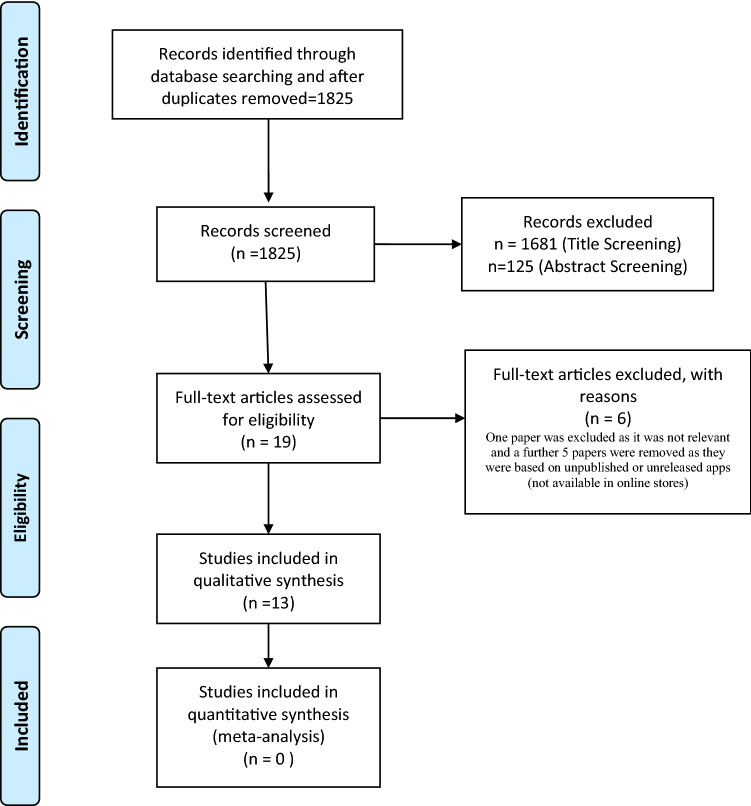
PRISMA

This systematic review of world literature was performed in the Cochrane style and in accordance with the Preferred Reporting Items for Systematic Reviews and Meta-Analyses (PRISMA) checklist (Fig. 1) [10, 11]. The search was for English language articles between inceptions of databases to June 2019, with the final search being conducted on 17/06/2019. Medline, EMBASE, Cochrane Library, CINAHL, and Web of Science were the databases searched. The search terms used were ‘prostate’, ‘cancer’, ‘prostate cancer’, ‘PSA’, ‘prostate specific antigen’, ‘prevention’, ‘adenocarcinoma’, ‘prostatic intraepithelial neoplasia’, ‘social media’, ‘phone app’, ‘apps’, ‘search engine’, ‘online’, ‘web-based’, ‘ehealth’, ‘mhealth’, ‘user-generated content’, ‘mobile health’, ‘smartphone’, ‘mobile phone’, ‘personal digital assistant’, ‘google play’, ‘android’, ‘apple’ and ‘iOS’. Medical Subject Heading (MeSH) phrases included ("Prostatic Neoplasms"[MeSH]) AND “Mobile Applications” [MeSH]); (‘‘Prostate’’ [MeSH]) AND ‘‘Smartphone application’’[MeSH]); (‘‘Prostate cancer’’[MeSH]) AND ‘‘Social media’’[MeSH]) AND ‘‘Mobile health’’[MeSH])aq. Boolean operators (AND, OR) were used to refine the search. Two reviewers (EJ and BS) identified all studies and those that appeared to fit the inclusion criteria were included for a full review. Papers evaluating a ‘general’ cancer app, that specifically mentioned prostate cancer patients within the article, were included. However, papers solely evaluating apps that did not have a cancer focus (e.g. pedometers or fitness trackers, without a cancer-related component) were excluded. Each reviewer independently selected studies for inclusion in the review and discrepancies were resolved by mutual consensus. A literature search had been run on each database. Any duplicates were excluded. At initial screening articles were excluded by title screening. The abstracts of the remaining articles were further screened and excluded if considered unsuitable for the review. Full text of the remaining literature was then reviewed. After a complete evaluation of the full articles, articles were excluded if deemed unsuitable. The remaining studies were included in the review for a narrative synthesis. The following information was extracted and organised using a spreadsheet (to perform further analysis): the year of publication, journal, number of applications assessed, type of apps and assessment criteria used on the included applications. References for these studies were collected using EndNote Web, and citations were either imported directly or manually entered. The outcomes of individuals studies will be presented in narrative fashions with emphasis on App development, App Readability, Quality and Accuracy of Apps, App Usage, and Risk Calculators.

### Review of mobile phone applications (apps)

The full-text articles were also assessed in detail for the apps and data was extracted for the following information (where available): name of application, developer, platform (Google Play Store or Apple App Store), and factors assessed by article. One of the authors (EJ) gathered further information on these apps which had been named by studies using the online stores (namely Google Play and App Store). This data included: the app’s cost, star rating [1–5], number of reviews, date of the last update, and the advertised content of the app (found in the app’s description). All information gained from both the online stores and from the literature was then compiled into the same spreadsheet for analysis. Data were collated using Microsoft Excel (version 12.2.4).

## Results

### Study selection

The initial literature search yielded 1825 results; 449 from Medline, 539 from EMBASE, 159 from CINAHL, 188 from Cochrane Library, and 490 from Web of Science. One thousand seven hundred and fifty-two articles were screened after removal of duplicates. 1608 articles were removed after title screening. 125 articles were removed after the abstract screening. Full texts of 19 studies were assessed. Of these, one paper was excluded as it was not relevant and a further five papers were removed as they were based on unpublished or unreleased apps (not available in online stores). This left 13 papers for inclusion in our final review [12–24] (Fig. 1).

### Description of studies

The papers included in this review focused on a variety of factors in assessing available apps including readability, quality and accuracy, usage, and app development. A comprehensive summary of the individual studies has been presented in Table 1. The authors have highlighted the outcomes of individual studies under the headings:

1. Studies related to app development.
2. Studies related to readability.
3. Studies related to ‘quality and accuracy’ of apps.
4. Studies related to app usage.
5. Studies related to risk calculators.

**Table 1:** Summary of included studies

Study	Study type	Objective	Outcome/conclusion
Brouard et al. [14]	Evaluation of oncological apps, and identification of relevant apps	Analysis of oncology apps to define content, assess business model, assess involvement of pharmaceutical industries, and determine scientific validation	The study identified 539 apps, with the majority dedicated to healthcare professionals, then to general population and then to patients
			The majority of apps concerned all types of cancer, with 26 being prostate focused
			Of the apps included, 36.5% had scientific validation mentioned in app descriptions, but this was less frequent for apps targeted at patients or the general population
			The majority of apps available had a focus on education as their main objective
			Authors concluded that patients and healthcare professionals should remain cautious about applications’ contents, and that there is a greater need for scientific validation
Hälleberg-Nyman et al. [16]	Evaluation of usage of a prostate cancer app (Interaktor) and patient participation	To explore how patients with prostate cancer perceived their own participation during radiotherapy, with or without the app	The paper found that patients perceived that participation in their care was greater when using an app, even though there wasn’t a difference in perception of satisfying basic needs
			Patients using the app felt that it facilitated participation (mutual participation in particular)
			Authors determined that using an app to maintain symptom management and provide contact between patients and health-care staff, can help increase patient participation in care
		Participation was explored in four dimensions: mutual participation, fight for participation, requirement for participation and participation in getting basic needs satisfied	
Langius-Eklöf et al. [18]	Evaluation of the uses of a prostate cancer app (Interaktor)	To investigate user behaviour, adherence to reporting, and patient experience of using a cancer app during radiotherapy for localised advanced prostate cancer	The app was found to Increase symptom reporting adherence, which allows clinicians to determine the most commonly reported symptoms and helps to determine the potential of radiotherapy to improve symptom burden
			Increase participants sense of security in their own well-being
			Act as a supportive tool for symptom self-management during prostate cancer treatment
Pereira-Azevedo et al. [20]	Development of a prostate risk-calculator app	To present the Rotterdam Prostate Risk Calculator app	The risk calculator uses PSA level, previous negative prostate biopsy, digital rectal examination (DRE) findings, prostate volume measurement, transrectal ultrasonography findings, MRI results, and Prostate Health Index to estimate overall and significant prostate cancer risk The app was found to
			Be useful in predicting the risk of prostate cancer, and of clinically significant cases
			Have usefulness of 92%, information quality of 87% and interface quality of 89%, when tested by participants
		Development and assessment of a smartphone app for prostate cancer screening, based upon the Rotterdam Prostate Cancer Risk Calculator	
Sundberg et al. [22]	Evaluation of usage of a prostate cancer app (Interaktor) in symptom management and detection during radiotherapy	Evaluation of the effects of symptom burden and quality of life when using the application for real-time symptom assessment and management during radiotherapy	It was found that the group using the app reported significantly lower levels of fatigue and nausea at the end of radiotherapy
			The app group had significantly less burden in emotional functioning, insomnia, and urinary-related symptoms at the end of treatment and 3 months later than the control group
			Authors found that the app (which they developed) had a role in facilitating supportive care needs during cancer treatment
		Performed using non-randomised control trial	
			This highlights the importance of early detection and management of symptoms (especially in anxiety and depression which can cause sleep disturbances), which can be facilitated by the use of the app
		Outcomes measured using ‘EORTC QLQ-C20′ and ‘Sense of Coherence questionnaire’	
Pham et al. [24]	Trail Design for Qualitative study to evaluate NED	Adoptability and acceptability by patients, caregivers, and clinicians	NED access given to 400 patients, 200 caregivers, and 10 clinicians
			Trial anticipated to have been completed (May 2019)
			Outcomes of this trial will improve understanding of the impact of PCA’s such as NED on prostate cancer survivorship programmes
Adam et al. [12]	Systematic review of prostate cancer risk calculator mobile apps	To review, rate and assess the everyday functionality and utility of all of the currently available prostate cancer risk calculator apps	Seven apps were critically appraised; 3 were exclusively android, 2 Apple, and 2 were available on both platforms The top-performing apps were found to be Rotterdam, then Coral, and then the CPC Risk Calculator
			The accuracy of all included apps was deemed acceptable
		Used uMARS (‘user’ version of Mobile Application Rating Scale) to assess app quality, subjective quality, and perceived impact	
Böhme et al. [13]	Evaluation of the quality and accuracy of mobile cancer apps	To evaluate the quality of mobile cancer apps	Of the 41 apps assessed, 6 scored ‘very high’, 15 scored ‘high’, 17 ‘deficient’ and 3 ‘insufficient’; therefore nearly half of the apps tested were deemed ‘deficient’ or ‘insufficient’, with the slight majority (51.19%) deemed ‘high’ or ‘very high’
		Developed a rating tool for assessing cancer apps using MARS (Mobile App Rating Scale), and GCS (German Cancer Society) instruments	
			Apps dedicated to/targeted at patients were better quality overall than others, and the group with the worst quality apps were the general population Apps deemed ‘deficient’ or ‘insufficient’ had particularly poor ratings e.g. in the sub-scales ‘information on sources’ and ‘data protection’
			Author raised concerns over data protection, as more data is now being required from users, and this may become of higher importance in the future, and suggested that there is need for improvement in regulation
		Assessed 41 apps of mixed cancer types (including breast, colorectal, prostate, gastrointestinal and general cancer apps), of which 18 were ‘general’ or prostate cancer specific	
		Considered quality of apps for target group (patients, general population, healthcare professionals)	
Røder et al. [21]	Development, validation and presentation of a prostate risk-calculator app	To present the CPC Risk Calculator app	The app was developed for use on both Android and iOS platforms
			App development involved usage of preoperative PSA, pTstage, prostatectomy Gleason score, and surgical margin (R) status
		Development and validation of a risk calculator that detects the absolute risk of biochemical recurrence following radical prostatectomy in men with an undetectable PSA	
			The app was found to Be accurate (70–85%)
			Predict risk of biochemical recurrence up to 12 years after radical prostatectomy
			Account for known risk-factors and other-cause mortality
Zhang et al. [23]	Presentation and development of a cancer app	To present the MyHealthAvatar app for breast and prostate cancer patients	The app was tested on user experience and visual design
			Early developmental flaws were outlined
			Feedback from testers showed that the app
		App was designed to facilitate health and lifestyle data presentation and analysis, and provide information to patients to aid with disease management	
			Improved user knowledge about their disease, and provided tailored information
			Improved users engagement in health and fitness activities, and raised user’s risk awareness in relation to their disease
			Was slow-loading (addressed by authors in the paper, as an area for future improvement)
De Nunzio et al. [15]	Performance and accuracy of prostate cancer risk calculator apps	Tested diagnostic performance and usability of 2 apps (Rotterdam Prostate Cancer Risk Calculator app, and Coral app) in patients at increased risk of prostate cancer, that were undergoing prostate biopsies	Authors found that the Rotterdam app outperformed Coral app in predicting prostate cancer and high-grade prostate cancer (0.7 vs. 0.631, and 0.75 vs. 0.69)
			Both apps were determined comparable in terms of usefulness (both > 80%), information quality (> 70%), interface quality (> 70%) and satisfaction (> 75%)
			54% preferred the Rotterdam app, and 46% preferred Coral
			Authors concluded that apps are outperforming website applications due to their better immediacy, compatibility, shareability and upgradeability
Kim et al. [17]	Readability and patient comprehension of cancer-related mobile apps	Analysed apps using readability studio software, over 10 readability assessments	Data specifically provided on prostate cancer apps
			‘Cancer Conditions and Treatments’ app was found to have a mean reading score of 10.2 (mean of all apps reviewed was between 9.0 and 14.6), therefore although the reading level is that of GCSE-level, it is more accessible than many of the other apps reviewed
			‘itsaMANTHING’ app had a grade-level readability of 9.1
			‘Mens Health Facts And Tips’ app was found to have a mean reading score of 10.6, therefore although the reading level is between GCSE and college-level, it is more accessible than many of the other apps reviewed
			‘Prostate Cancer’ app (by developer ‘Focus’) had a grade-level readability of 9.5 (GCSE level)
			Authors stated that the prostate cancer apps (and all others included in the study) had high reading levels that preclude understanding in the average patient
			Found that only 2 of the 21 applications (not specified which) were developed by someone with an ‘adequate’ background in medicine or science
			Concluded that clinicians may need to recommend apps with easier readability to their patients
		Analysed apps relating to several cancers (lung, breast, colorectal, gastric and prostate)	
		21 apps were included	
Owens et al. [19]	Systematic review of prostate cancer apps	To identify and evaluate apps which promote informed prostate cancer screening decisions	Data specifically provided on prostate cancer apps
			12 apps contained accurate information about anatomy and function of prostate, prevalence and incidence of prostate cancer
			Eleven apps included accurate information about risks and symptoms
		Fourteen apps were identified through the Apple App Store, and Google Play Store	
			Nine apps included information about screening ages
			Eight apps included accurate information about digital rectal examination
			12 apps included accurate information about the PSA, thirteen presented a neutral tone when discussing it and one was pro-screening
			Average reading was found to be of 10th grade level, with 4 at 8th grade level and 5 at 9th or higher
			Five apps did not meet any of the cultural sensitivity criteria implemented by the authors
			Eleven apps focused on providing general information
			Recommendations by paper:
			Apps should include information consistent with latest evidence
			Culturally sensitive language should be used
			Developers should be aware of implications of framing of content (e.g. as pro or against screening)
			Apps should be interactive and useable
		Evaluated whether apps provided information about the location and function of the prostate; prevalence, incidence, symptoms, and risks of prostate cancer; information about recommended screening age, digital rectal exam and PSA	Apps should be developed collaboratively (with healthcare/medically trained professionals)
		Assessed accuracy and breadth, framing of the prostate cancer screening controversy, grade-level readability, cultural sensitivity, and usability	

### Studies related to ‘app development’

Four studies focused on the development and testing of apps [18, 20, 21, 23]. Zhang et al. [23] presented the MyHealthAvatar app. This was a European Commission funded research project for patients with prostate and breast cancer. The app is available on desktop, tablet and smartphone. The app encourages patient self-management of their disease. It contains lifestyle and activity tracking. It has prostate and breast cancer questionnaires to monitor progress after treatment. It also provides advice on pelvic floor exercises for patients who have had a radical prostatectomy [23, 25]. The app includes International Index of Erectile Function-5 (IIEF-5) and the International Prostate Symptom Score (I-PSS) questionnaires and resources from the Prostate Cancer UK and NHS, UK. They also tested user experience and outlined the early developmental flaws. Data from this app can support research activity.

Røder et al. [21, 26] developed and validated the CPC Risk Calculator which estimates the risk of biochemical recurrence following a radical prostatectomy. The authors used preoperative PSA, pT stage, prostatectomy Gleason score, and surgical margin (*R*) status to develop the nomogram. The data was calculated from 2167 men who underwent a radical prostatectomy at the Copenhagen Prostate Cancer Center, Rigshospitalet, Denmark. The nomogram was externally validated using a cohort of 2237 men who underwent a radical prostatectomy at the Stanford University, California, USA in the same time period. The authors reported high accuracy and discrimination on external validation. The accuracy of model declined after 7 years due to limited follow-up in the 2 cohorts.

Pereira-Azevedo et al., evaluated their app, the Rotterdam Prostate Cancer Risk Calculator, via usability testing [20, 27]. Rotterdam Prostate Cancer Risk Calculator is developed using algorithms from Rotterdam arm of the European Randomized Study of Screening for Prostate Cancer (ERSPC) study. The calculator uses PSA level, previous negative prostate biopsy, digital rectal examination (DRE) findings, prostate volume measurement, transrectal ultrasonography findings, MRI results, and Prostate Health Index to estimate overall and significant prostate cancer risk. The app was scored by participants, on usefulness, quality of information, and quality of interface scoring highly (gaining ≥ 87%) in all categories.

Langius-Eklöf et al. [18] tested their app, Interaktor, on prostate cancer patients undergoing radiotherapy, to determine its potential to ease symptom burden. They found high adherence to symptom reporting and realised a novel use for the app for clinicians, to determine the most commonly reported clinical side effects of their patients.

All four papers found that users benefited from, or had a neutral experience when using their apps, and authors explored potential possibilities for these apps to benefit healthcare professionals as well as patients (who the apps were designed for) [20, 22–25].

Pham et al., reported on a trial design to evaluate the acceptability of NED (No Evident disease) by patients, caregivers and clinicians. NED is a prostate cancer app developed to support prostate cancer survivorship programmes [24].

### Studies related to ‘app readability’

Kim et al. [17] and Owen et al. [19] tested the readability of cancer apps that are currently available online. Both papers used readability assessment tools to determine the grade-level readability of each app. Kim et al. evaluated readability of PCAs along with four other cancer apps available on Apple Store and Google Play Store. The study identified 12 articles from 3 PCAs (Mens Health Facts and Tips, ProstAid, Prostate Cancer) for evaluation. The study reported that PCAs available on Apple Store and Google Play Store had average reading grades of 10.6 and 9.4 respectively [17]. Owens et al. identified 14 PCAs. 10 PCAs had adequate material for readability evaluation with the average reading to be at 10th grade level [19]. The study concluded that the apps included in the study were of high reading levels that were greater than that of the average patient, which might prevent patients from understanding the information they contain.

### Studies related to ‘quality and accuracy of apps’

Owens et al. [19] also explored the quality of content provided by 14 PCAs. This was based upon accuracy, breadth, tone/framing and cultural sensitivity of the app’s content. Best Prostate Cancer Treatment, Oncotip, and Prostate Cancer by Magna Health Solutions were the 3 PCAs that had most extensive detail on prostate cancer covered. Authors found that 13 of the 14 apps studied had a neutral tone with regards to PSA testing. Oncotip was the only PCA that was pro-screening. Overall, the majority of apps tested provided an accurate and wide range of information and were of good quality. The overall rating of the ‘14 PCAs for cultural sensitivity for African Americans was low. Procee had the best rating for cultural sensitivity [19].

Bohme et al. [13] evaluated the quality of apps, using the Mobile Application Rating Scale (MARS) and German Cancer Society (GCS) instruments to determine the quality of information contained within the apps for breast, prostate and colorectal cancer. The tools had 3 domains (engagement, aesthetics and information) and 22 aspects were evaluated. 24 apps in the study were PCAs. Of all apps included in the study, around 48.78% were considered deficient or insufficient [13]. The authors reported the overall quality of apps which were targeted at patients were of better quality than those targeted at either healthcare professionals or the general population. The study highlighted deficient or insufficient scores for data protection.

### Studies related to ‘app usage’

Three papers examined the usage of PCAs [14, 16, 22]. Hälleberg-Nyman et al. [16] and Sundberg et al. [22] both assessed the app ‘Interaktor’ and determined it to be a useful tool. Sundberg et al., found that when using the app for the real-time assessment of symptoms in prostate cancer patients undergoing radiotherapy, the control group (who did not use the app) displayed significantly worse emotional functioning at the end of radiotherapy when compared to the intervention group. Authors posited that this highlights the importance of the early detection and management of symptoms—something which apps can facilitate [22]. Furthermore, Hälleberg-Nyman et al. [16] found that patients using the app had a greater perceived participation in their care, which may be important in doctor–patient relationships and patient outcomes.

Brouard et al. [14] found that the majority of apps available had a focus on education as their main objective. They also found that the apps included in their study which were aimed at patients or the general population had less scientific validation than those targeted towards healthcare professionals [14].

### Studies related to ‘risk calculators’

Two papers solely explored risk-calculator applications and their accuracy [12, 15]. De Nunzio et al., compared the performance of Rotterdam [27] and Coral [28] in 1682 patients undergoing prostate biopsies for suspected prostate cancer. Rotterdam was significantly better than the Coral at predicting overall (AUC: 0.70 versus 0.631, *p* = 0.001) and high-grade prostate cancer (0.75 versus 0.69, *p* = 0.001). Both apps were accurate and comparable in terms of usefulness (both > 80%), information quality (> 70%), interface quality (> 70%) and satisfaction (> 75%) [28]. However, 54% of participants preferred Rotterdam overall. Rotterdam also was deemed the best by Adam et al., who critically appraised 7 applications across both Android and iOS platforms [12, 15]. They found that the top-performing apps when using the uMARS scale (user version of the MARS scale) to assess quality, were Rotterdam, then Coral, and then the CPC Risk Calculator, although accuracy of all included apps was deemed acceptable [12].

### Individual applications includes studies evaluated by the authors

Of the 12 papers included in the study, 44 apps were identified for which we collected data. Due to the rapidly developing nature of applications, at the time of the study 11 apps were no longer available to evaluate. The remaining 33 apps were mainly free, with only 2 requiring a subscription. As found by authors of the papers included in this review, the majority (59%) of the apps had an educational focus, with other objectives being risk assessment, support, or targeted towards clinicians for information or decision making. Interestingly only 5 of the apps had been updated within the last year, which may suggest that information within the other apps may not entirely up to date. The full analysis and breakdown of these apps and their content can be seen in Table 2.

**Table 2:** App content and analysis

App content	App	Platform	Cost	^a^Rating	Number of reviews	Latest update	Developer	Platform description
Education—prevention (for the general public)	300 tips to prevent cancer	Google Play Store	Free	4.8	68	28/10/2018	Let ME Hear Again Apps	Provides daily health tips and articles on healthy foods
								Lifestyle tips to prevent cancer and recurrence
	Cancer awareness	Google Play Store	Free	0	0	03/02/2014	Surendrasinh Champavat	Provides information about prostate cancer e.g. regarding radiation, chemotherapy, and prevention
	Cancer Conditions and Treatments	Google Play Store	Free	5	3	08/11/2013	Space-O Infoweb, Inc	Provides information about signs, symptoms, diagnosis, treatment, statistics and risk factors
	Cancer Research News & Prevention Info	Apple App Store	Free (Pro is $3.86)	5	1	19/11/2016	Juicestand Inc	Provides users with latest cancer research news and prevention information
								Includes videos
	PCFA Know Your Score WA	Apple App Store	Free	0	0	25/01/2017	CommunityToGo Pty Ltd	Educates and inform users about prostate cancer
								Engages users with competitions
	Prostate Cancer	Google Play Store	Free, but $9.02 for in-app purchases	3	1	28/10/2017	Focus Medica India Pvt. Ltd	Improves user understanding using animated videos
								Educates users on the anatomy of the prostate, and the symptoms, causes, risk factors, staging and prognosis etc. of prostate cancer
	Prostate Cancer	Google Play Store	Free	4.3	15	22/03/2017	Anastore	Provides information about causes, symptoms and statistics regarding prostate cancer
	Prostate cancer	Apple App Store	Free	0	0	04/03/2017	Magna Health Solutions	Provides information about causes, symptoms and treatment of prostate cancer
	Prostate Pal 3	Apple App Store	Free	5	1	06/05/2015	Ronald L. Yap, M.D	Helps men track their prostate health
								Includes a bladder diary and PSA tracker
	^a^PROCEE	Google Play Store	U	U	U	U	Interactive Systems Research Group	No longer available
	^a^Prostate Cancer Treatment and Prevention	Apple App Store	U	U	U	11/07/2016	Monica G	No longer available
	^a^iCancer health: cancer care–virtual care at home	U	U	U	U	U	U	No longer available
	^a^Cancer Support	U	U	U	U	U	U	No longer available
	^a^Zero Prostate Cancer News	Google Play Store	U	U	U	U	Fuzz Labs	No longer available
Education—for cancer patients	^a^ADT	Apple App Store	Free	U	U	U	Jim Duthie	No longer available
	CancerAid—empowering cancer patients and carers	Google Play Store	Free	3.7	25	01/05/2019	CancerAid PTY LTD	Provides patients with medically reliable information
								Helps patients track treatment information, symptoms and medication use
	Focalyx	Apple App Store and Google Play Store	Free	5—Google Play Store 0—Apple App Store	6—Google Play Store 0—Apple App Store	18/11/2018	Lyx Health	For monitoring the diagnostic and treatment characteristics of men diagnosed with prostate cancer
								Allows for patient and physician interaction in-app
	Best Prostate Cancer treatment	Apple App Store	Free	0	0	22/09/2017	RL Technology, LLC	Provides ‘natural’ treatments
								Provides videos on how to ‘assist and cure’ cancer
	itsaMANTHING	Apple App Store and Google Play Store	Free	5—Google Play Store and Apple App Store	5—Google Play Store 1—Apple App Store	09/02/2015—Google Play Store 26/01/2015—Apple App Store	PROSTaid	Provides information about prostate cancer symptoms, appointments, diagnosis, treatments
								Written by patients, for patients
	Mens Health Facts and Tips	Apple App Store	Free	2.4	2	14/08/2014	Michael Quach	Provides users with information regarding screening (also suitable for medical professionals, students, and the general public)
	My Prostate Cancer Manager	Apple App Store	Free	0	0	16/01/2019	@Point of care	Helps patients manage symptoms, track their progress, manage medication and treatment, and share their symptoms with healthcare providers
	My Prostate Health Navigator	Apple App Store	Free	0	0	10/10/2015	Sourcetoad, LLC	Provides up-to-date medical information and resources related to prostate cancer
								Users can interact with other patients, physicians and watch videos
								For the general public too
	MyHealthAvatar	Google Play Store	Free	5	2	22/08/2018	AnSmart	Aids patients in monitoring their daily health e.g. activity tracking, mood tracking, medication tracking
	Prostate Cancer Support Group Gibraltar	Apple App Store	Free	0	0	22/10/2016	Alan Pereira	Provides information, support and counselling to those affected by prostate cancer
	Prostate Cancer Treatment	Google Play Store	Free	0	0	22/10/2018	Creative Live Apps	Provides information about prostate cancer treatment, treatment side effects, and staging
	NCCN Patient Guides for Cancer	Apple App Store and Google Play Store	Free	4.5—Google Play Store 0—Apple App Store	4—Google Play Store 0—Apple App Store	07/06/2017	National Comprehensive Cancer Network (NCCN)	Easy-to-understand resources for patients, based upon clinical guidelines
								Summary of key points and glossary for patients
	^a^Interaktor	U	U	U	U	U	U	No longer available
Education – for professionals and students	Cancer mAPP	Apple App Store	Free	5	3	02/10/2016	Scott Berry	A database of summaries from hundreds of clinical trials
	iURO Oncology	Apple App Store	Free	5	1	26/02/2016	CommunityToGo Pty Ltd	Contains narrated simulation videos to improve understanding of prostate cancer pathologies and therapies
	Wallpaper of the Salvador Gil Vernet Collection of Urology Drawings	Apple App Store and Google Play Store	Free	0	0	12/11/2017	eldeAM- Google Play Store Josep Solanes Batllo, BlueBOARD—Apple App Store	Provides a selection of urology drawings from the Salvador Gil Vernet Collection—including gross anatomy, urogenital pathology, and surgical techniques
	^a^Cancer News Reader—research, drug directory, alternative treatments etc	U	U	U	U	U	U	No longer available
	^a^Cancer Screening	U	U	U	U	U	U	No longer available
	^a^Prostate Cancer MiMe	Google Play Store	Free	U	U	15/07/2016	e-HIMS bvba	No longer available
Screening—for clinicians	Cancer Genetics	Apple App Store and Google Play Store	Free	3.7—Google Play Store 5—Apple App Store	3—Google Play Store 5—Apple App Store	18/02/2016—Google Play Store 05/02/2016—Apple App Store	UBQO Limited	Provides risk assessments and referral guidance for hereditary cancers
	Coral—Prostate Cancer Risk and Survival	Apple App Store	Free	0	0	06/04/2017	Jon Giambattista	Provides clinical nomograms specific to prostate cancer to guide clinical decision making
	Prostate Cancer Calculator	Google Play Store	Free	3.6	22	29/01/2017	Bornifer LLC	Calculates international prostate symptom score
								Calculates PSA density, velocity and doubling time
								Calculates risk of biopsy-detectable prostate cancer
								Estimates the optimum number of prostate biopsy cores needed
	Rotterdam Prostate Cancer Risk Calculator	Apple App Store and Google Play Store	$1.92—Google Play Store $2.57—Apple App Store	4.5—Google Play Store 0- Apple App Store	10- Google Play Store 0- Apple App Store	24/04/2019—Google Play Store 10/04/2019—Apple App Store	Stichting SWOP, Nuno Azevedo	Provides a general risk calculation based upon PSA levels and other information such as MRI results
	Prostate Volume and Density	Apple App Store and Google Play Store	Free	4.8—Google Play Store 0—Apple App Store	5- Google Play Store 0- Apple App Store	16/04/2017	iMedical Apps- Google Play Store Putu Angga Risky Raharja—Apple App Store	Helps healthcare professionals assess patients with enlarged prostates, by calculating the volume and density of patients’ prostates
	^a^PSA Calculator	Google Play Store	$1.92	U	U	U	Peterson Leite	No longer available
Screening—for patients and the general public and/ or clinicians	Capra Score	Apple App Store	Free	0	0	26/04/2017	Phillip Dorch, MD	Calculates CAPRA score for patient with prostate cancer
	NED	Apple App Store Google Play Store	Free	U	0		University health network	Prostate Cancer survivorship App
								Sends automated notifications to clinicians about PSA and recorded symptoms
								Reminders to complete wellness and quality of life questionaries’
	Capra-S Calculator	Apple App Store	$1.28	0	0	26/04/2017	Phillip Dorch	Assesses the risk of prostate cancer recurrence after first-line surgery, and provides predictions at 3 and 5 years post-surgery
	IPCRC	Google Play Store	Free	5	65	18/02/2016	Prahara Yuri, fath2app	Provides risk calculation based on age, PSA, prostate volume and DRE findings
	CPC Risk Calculator	Apple App Store and Google Play Store	Free	0	0	30/11/2016	Daman P/S	Estimates risk of biochemical recurrence after radical prostatectomy

^a^App not available on current versions of platforms; U; data unavailable; Cost in USD; converted from GBP on 07/02/20 (1GBP = 1.29USD)

## Discussion

This systematic review has identified 44 PCAs targeting the general population, patients and clinicians, with a majority (33 of 44) of them focusing on education. It is the authors view that existing PCA’s are currently in its infancy and do require further development before widespread integration into existing clinical practise. The apps covered topics such as lifestyle changes, and information on prostate cancer including treatment options, PSA screening, symptomatology, diagnostics, statistics, research and prostate anatomy. Three prostate cancer risk calculators (Rotterdam, CORAL and CPC) were identified which provided estimates on prostate cancer diagnosis and biochemical recurrence following radical prostatectomy. Rotterdam was the best performer amongst the 3 risk calculators. Most PCAs were rated to have a high standard of readability, raising concerns that a proportion of the patient population may not be able to adequately comprehend the available information in them. Additionally, one study reported deficient or insufficient quality for data protection for cancers apps [13]. The gross majority (36 of 43) of the PCAs haven’t been updated in the last year and, therefore, there is doubt if the existing data in these apps is current.

PCAs have the potential to have a number of roles in the contemporary management of prostate cancer. Healthcare organisations world-wide have adopted the principles of shared care decision making (SDM) between a healthcare professional and patients [29, 30]. PCAs, in addition to existing Decision Aids (DA) can be useful adjuncts to clinical counselling, facilitating well-informed clinical decision making, improve clinician-patient communication and as a consequence leading to a favourable patient experience [29]. PCAs such as Interaktor and MyHealthAvatar (MHA) are such PCAs that have been developed as supportive aids that compliment clinical consults. Hälleberg-Nyman et al. [16] in qualitative study reported patient-reported satisfaction scores to be better in patients receiving radiotherapy, when clinical consults were supplemented with the interactive app, Interaktor, corroborating the aforementioned view. In prostate cancer, SDM with DAs is particularly pertinent, due to controversies in areas such as prostate cancer screening and the availability of plethora of therapeutic options [31]. In this review, a number of PCAs addressed the subject of PSA screening and reassuringly most PCAs had a neutral tone for PSA screening. PCAs can be useful adjuncts to clinical consults in this context, conforming to the principles of informed patient choice and avoiding decision regret.

Prostate cancer diagnostics has seen significant evolution in recent years with strategies such as pre-biopsy multi-parametric MRIs [32]. This trend is likely to continue with the pursuit for biomarker technologies in prostate cancer [32]. Assimilation and presentation of ever-growing data from existing and novel diagnostic tools, in short clinical consults can be challenging. PCAs such as Rotterdam and CORAL integrate data from diagnostic tools and demographics, subsequently presenting an estimated risk of prostate cancer [27, 28]. PCAs such as Rotterdam and CORAL are hence invaluable aids to clinicians, allowing for seamless, efficient and accurate patient counselling. However, it is important to note that whilst Rotterdam does include MRI results as a criterion, CORAL does not, which may affect the accuracy of the result. Similarly, biochemical recurrence predictions following curative local treatments can be challenging and CPC calculators are therefore useful tools for clinicians [26]. Innovative Prostate cancer survivorship programmes will be required to manage an increasing population of prostate cancer survivors. Chu et al. [33] in a retrospective review reported over 95% patient satisfaction rates and individual patient savings of 193 US dollars with telemedicine delivered care. PCAs such as NED lineate well with prostate survivorship programmes and can be employed for post-treatment surveillance without the need for periodic attendance at hospital. This has benefits to patients living in remote locations with poor health care accessibility and also cost-saving benefits to healthcare organisations and individual patients.

Predictive analytics is increasing being adopted in healthcare to improve operational efficiency and disease management [34]. Studying behavioural and lifestyle patterns across a wide range of demographics can facilitate the identification of causal relationships. Medical apps are a useful information communication technology for large volume real-world data collection mitigating some of the challenges of traditional data collection. MyHealthAvatar (MHA) is PCA that has the ability to collate demographic, behavioural, lifestyle, and medical data for prostate cancer patients [23, 25]. These allow for analysing data in multiple clinical scenarios and can, therefore, lead to the creation of various virtual patient populations. These provide invaluable data to healthcare providers which may contribute to future stratified individualised care [35].

Despite the potential benefits of medical health apps in general and PCAs specifically, the potential for harm is real. PCAs must be accurate, easily comprehendible, un-biased and regularly updated. This review suggests that PCAs do not consistently fulfil all these pre-requisites. Brouard et al. [14] reported a majority of medical apps targeted at patients and the general population haven’t had scientific validation. Misinformation can lead to anxiety, over-diagnosis and over-treatment. It is therefore vital these apps are appropriately governed by stringent regulation to ensure patient safety. In Europe and the United Kingdom, current guidelines recommend only app with a CE marking are approved for clinical use [36]. Local institutions would be advised to have agreed on protocol of PCAs usage in clinical practise [36]. Bohme et al. [13] reported most cancer apps to deficient or insufficient in data protection and, therefore, caution must be exercised before patient sensitive information is added to these apps. Furthermore, healthcare professionals must be provided with formal education on the potential harms of modern day medical apps so as to ensure responsible usage.

Limitations of our study included the exclusion of grey literature, and papers not written in English. Although there are other sources of social media such as twitter, YouTube and google search engines, however in this paper we focussed on the telephone-based apps only. Due to the constant changes in the nature of apps, older software was not always updated and occasionally removed in time, hence our inability to find some of the apps mentioned in the papers analysed.

## Conclusion

There are a wide variety of PCAs available targeting the general population, patients and clinicians, with a majority of them focusing on education. The apps covered topics such as lifestyle changes, and information on prostate cancer including treatment options, PSA screening, symptomatology, diagnostics, statistics, research and prostate anatomy. A number of PCAs haven’t undergone scientific validation. There are concerns with data protection, high readability standards and lack of information update in current PCAs. There must be increased awareness among patients and clinicians about existing PCAs and their limitation so as to ensure safe and responsible usage. It is the authors view that existing PCAs are currently in its infancy and do require further development before widespread integration into existing clinical practise. If developed appropriately with responsible governance, they do have the potential to play important roles in modern day prostate cancer management.
